# Data on the expression of SRPK1a in mammals

**DOI:** 10.1016/j.dib.2019.104210

**Published:** 2019-07-03

**Authors:** Metaxia Vlassi, Konstantinos A. Kyritsis, Ioannis S. Vizirianakis, Thomas Giannakouros, Michalis Aivaliotis, Eleni Nikolakaki

**Affiliations:** a Institute of Biosciences & Applications, National Centre for Scientific Research “Demokritos”, Athens, Greece; b Laboratory of Pharmacology, Department of Pharmacy, Aristotelian University, Thessaloniki, Greece; c Laboratory of Biochemistry, Department of Chemistry, Aristotelian University, Thessaloniki, Greece; d Laboratory of Biological Chemistry, School of Medicine, Aristotelian University, Thessaloniki, Greece; e Functional Proteomics and Systems Biology (FunPATh)-Center for Interdisciplinary Research and Innovation (CIRI-AUTH), Thessaloniki, Greece

**Keywords:** SR protein kinase, SRPK1, SRPK1a, Mammals, Phylogenetics

## Abstract

SRPK1 is an evolutionary conserved protein kinase that specifically phosphorylates its substrates at serine residues located within arginine-serine-rich (RS) domains. We have previously reported the existence of a second less abundant isoform in humans, SRPK1a, which is formed from alternative splicing of the *SRPK1* gene and contains an insertion of 171 amino acids at its N-terminal domain (Nikolakaki et al., 2001). In the NCBI database SRPK1a is annotated as a related to SRPK1-mRNA sequence coding for protein CAC39299.1. Here, we present data on the conservation of the extra sequence of SRPK1a in mammals. Furthermore, the retrieved sequences were comparatively analyzed and data on their evolutionary origin and relationships are also presented.

**Table:** Specifications table

Subject area	*Biology*
More specific subject area	*Biochemistry, Computational Biology and Data mining, phylogenetic analysis, SR protein kinases*
Type of data	Tables, figures
How data was acquired	*SRPK1a nucleotide and protein sequences were retrieved from NCBI Nucleotide and Protein databases. RNA-seq data are publicly available in the Gene Expression Omnibus (GEO) repository. Mass spectrometry data are publicly available in the EMBL-EBI PRoteomics IDEntifications (PRIDE) database.*
Data format	Raw and analyzed
Experimental factors	*Nucleotide and protein sequences were retrieved from online databases and used for detection of sequence conservation and phylogenetic analysis*
Experimental features	*Sequence similarity searches and multiple sequence alignments were performed using SRA BLASTN, BLASTP and COBALT. The PeptideMass tool was used to retrieve in silico specific SRPK1a peptide sequences and match them with mass spectrometry data available from the PRIDE database. Evolutionary analyses and drawing of the phylogenetic tree were conducted using MEGA6*
Data source location	*Analyses were performed at the Aristotelian University, Thessaloniki Greece and the National Centre for Scientific Research “Demokritos”, Athens, Greece*
Data accessibility	Data are provided with this article

**Table:** 

**Value of the data** • The presented data support the expression of SRPK1a in a variety of mammalian organisms and therefore, they may boost future research probing into the contribution of SRPK1a to SRPK1 functions. • Protein sequencing alignments provide information on the highly variable N-terminal domain of SRPK1a. These data will feed into the development of specific tools and experimental approaches for the genetic and biochemical analysis of SRPK1a function. • Phylogenetic analysis provides information on the evolutionary history of the SRPK1 gene and may lead to further downstream studies.

## 1 Data

**Fig. 1:**
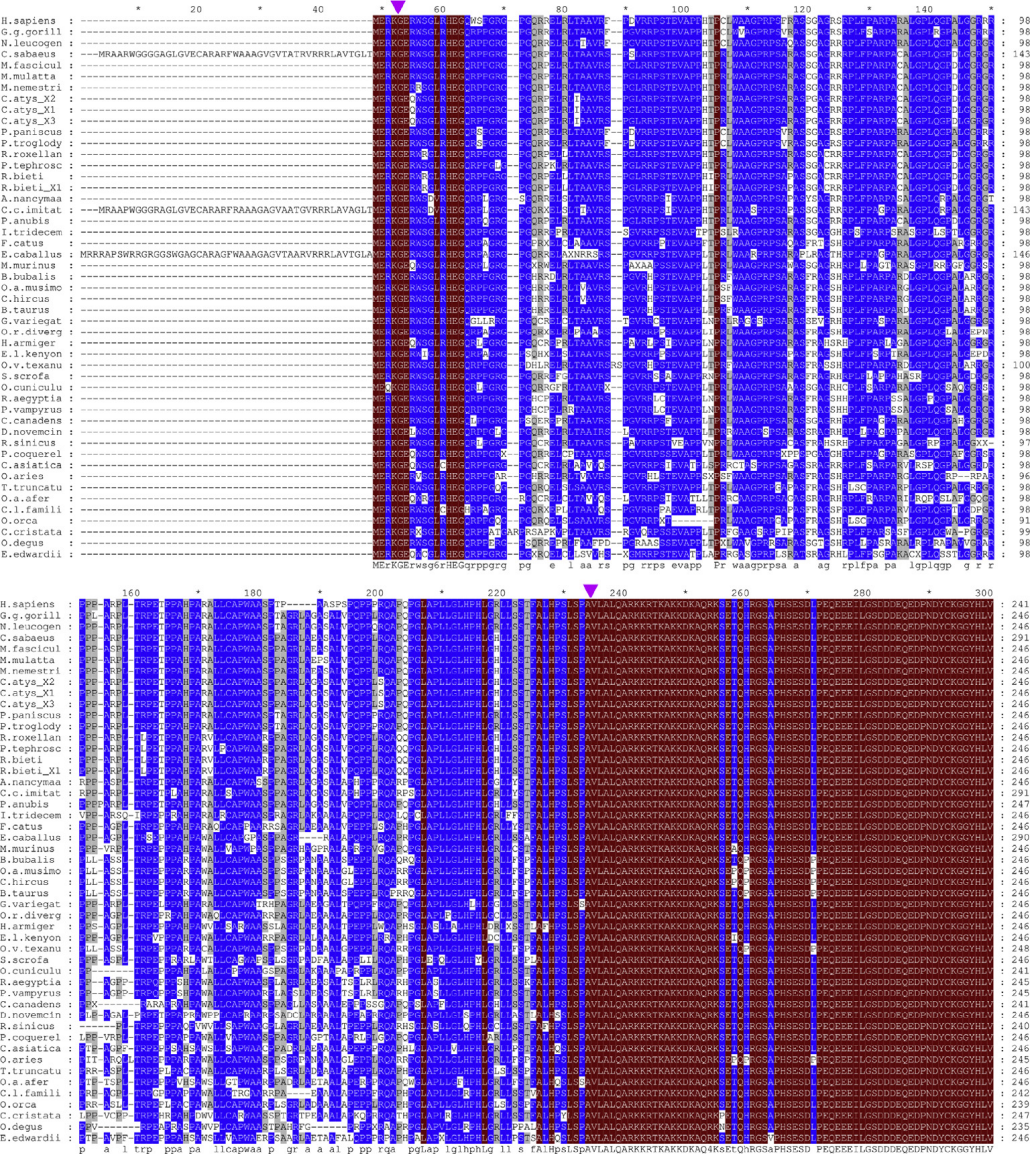
Conservation-based colored sequence alignment, drawn using the GeneDoc program [9]. Nomenclatures are according to organism names. The corresponding accession numbers in the NCBI protein database are shown in Fig. 2. Coloring scale: red, blue, and grey, for 100%, 80% and 60% sequence conservation, respectively. Arrows denote the starting and ending amino acid residues (glycine and alanine, respectively) of the additional sequence which is found only in SRPK1a and is omitted in SRPK1.

**Fig. 2:**
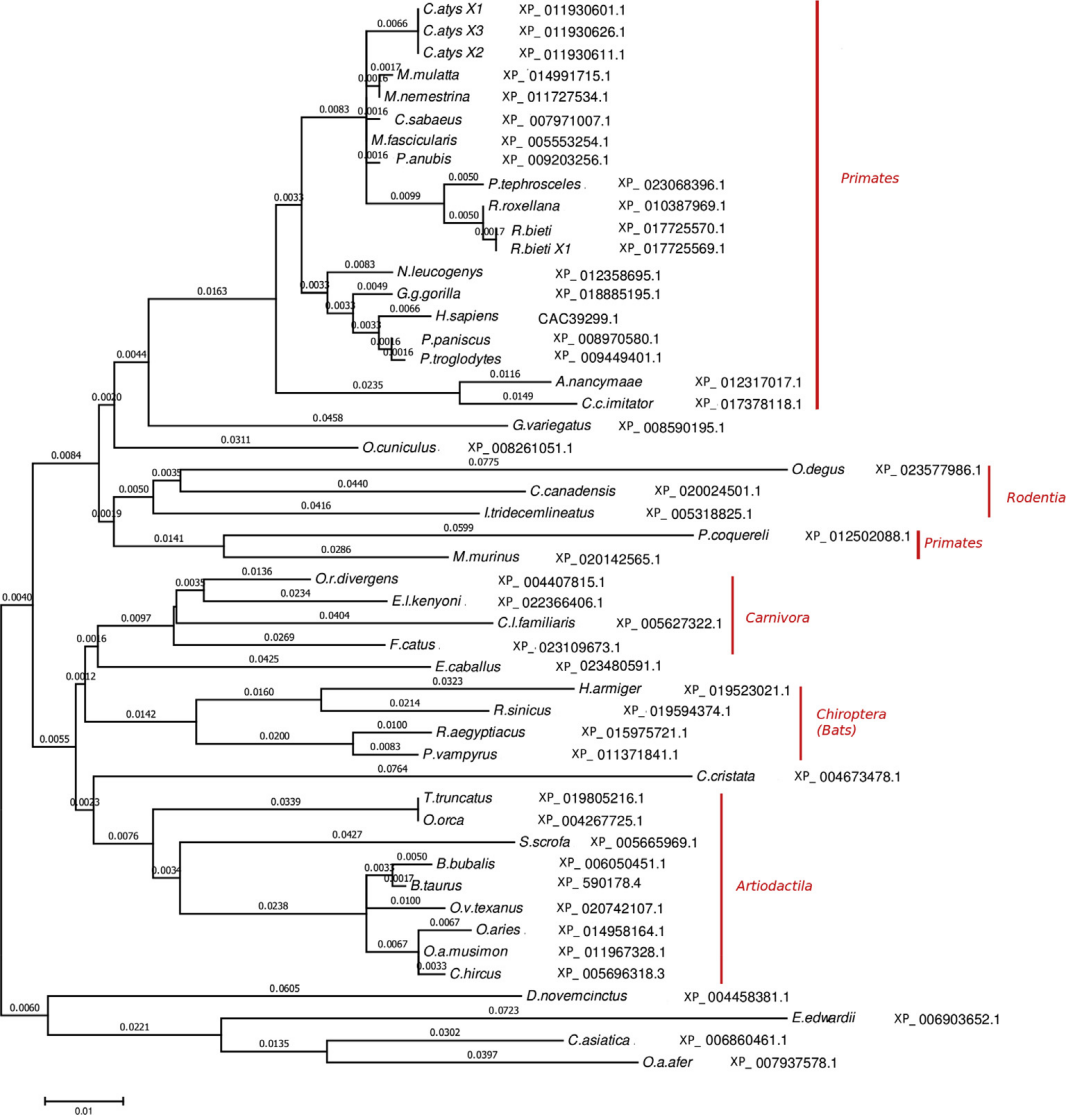
Molecular Phylogenetic analysis by the Maximum Likelihood method. The evolutionary history was inferred by using the Maximum Likelihood method based on the JTT matrix-based model [11]. The tree with the highest log likelihood (−6947.2720) is shown. Initial tree(s) for the heuristic search were obtained automatically by applying Neighbor-Join and BioNJ algorithms to a matrix of pairwise distances estimated using a JTT model, and then selecting the topology with superior log likelihood value. The tree is drawn to scale, with branch lengths measured in the number of substitutions per site (above the branches). The analysis involved 49 amino acid sequences (the corresponding accession numbers in the NCBI protein database, are indicated; see also Fig. 1). All positions containing gaps and missing data were eliminated. There were a total of 605 positions in the final dataset. Evolutionary analyses were conducted in MEGA6 [5].

Table 1 is an alignment of the additional SRPK1a mRNA 23-535nt region (Nikolakaki et al., 2001) [1], not found in SRPK1, against RNA-Seq reads of erythroid cell populations [2]. Fig. 1 is a conservation-based colored alignment of partial SRPK1a sequences that contain only the additional N-terminal part of the kinase, in mammals. Table 2 is a list of peptides originated from *in silico* trypsin digestion of SRPK1a 22-534nt translated region that returned a match in PRIDE database [3], [4]. Fig. 2 is a phylogenetic tree obtained by Maximum Likelihood method in MEGA6 [5].

**Table 1:** Local alignment of the 23–535 nt segment of SRPK1a mRNA (AJ318054.1), which corresponds to the retained intron that sets the difference between SRPK1a and SRPK1, against RNA-Seq reads of erythroid cell populations (GSE53635), using SRA BLASTN [6]. Raw RNA-Seq data are publicly available in the Gene Expression Omnibus (GEO) repository and can be accessed using experiment (GSE_ID) or sample (SRA_ID) Accession IDs.

Gene	Accession_ID	mRNA_region	GSE_ID	SRA_ID	Cell_type	No of_RNA-Seq_reads (100%)
SRPK1a	AJ318054.1	23–535	GSE53635	SRX398536	proerythroblast	27
SRPK1a	AJ318054.1	23–535	GSE53635	SRX398537	early_basophilic	7
SRPK1a	AJ318054.1	23–535	GSE53635	SRX398538	late_basophilic	6

**Table 2:** *In silico* trypsin digestion of SRPK1a 22–534 translated region. Below are shown the resulting peptides that returned a match in PRIDE database (PRD000004, human plasma proteome; PXD001383, chromatin-associated and soluble human transcription factor complexes; PXD000593, human CDK family protein complexes), as well as the local alignment identity score of SRPK1a (CAC39299.1) with the PRIDE peptide matches using BLASTP [7].

Gene	Accession number	*In silico* digestion position	Tryptic peptide	PRIDE database tryptic peptide match	PRIDE database Accession ID	BLASTP top result	BLASTP identity score (%)
SRPK1a	AJ318054.1	94–113	RPPPARPLTRPETPPAHPAR	RPPPARPLTRPETPPAHPARALLCAPWAASPPAASPPQPPPR	PRD000004	SRPK1a (CAC39299.1)	95
SRPK1a	AJ318054.1	138–156	QAPQPGLAPLLGLHPHLGR	RQAPQPGLAPLLGLHPHLGR	PXD001383	SRPK1a (CAC39299.1)	100
SRPK1a	AJ318054.1	157–171	LLSSTFALHPSLSPA	LLSSTFALHPSLSPAV	PXD001383	SRPK1a (CAC39299.1)	100
SRPK1a	AJ318054.1	80–92	ALGPLQGPALGGR	RALGPLQGPALGGR; ALGPLQGPALGGR	PXD001383; PXD000593	SRPK1a (CAC39299.1)	100

## 2 Experimental design, materials and methods

Sequence similarity searches and multiple sequence alignments were performed using the NCBI tools: Sequence Read Archive (SRA) BLASTN [6], BLASTP [7] and COBALT [8], respectively. The GeneDoc tool [9] was employed for conservation-based color visualizations of the alignments and production of the alignment figures. The PeptideMass tool in default settings [10] was used to retrieve *in silico* specific SRPK1a peptide sequences, following *in silico* trypsin digestion of the human SRPK1a mRNA (AJ318054.1) 22-534nt segment translated into protein (sequence included within the arrows in Fig. 1). The resulting peptides were then used as queries in the PRIDE database [3], [4]. Evolutionary analyses and drawing of the phylogenetic tree were conducted by the Maximum Likelihood method in MEGA6 [5]. Annotations of organisms were done manually, based on the known classification of the organisms described in the COBALT output.
